# Sleep Quality among Medical Students of a Tertiary Care Hospital: A Descriptive Cross-sectional Study

**DOI:** 10.31729/jnma.4813

**Published:** 2020-02-29

**Authors:** Nabin Sundas, Saransh Ghimire, Suzit Bhusal, Rakshya Pandey, Krishna Rana, Hemang Dixit

**Affiliations:** 1 Kathmandu Medical College, Sinamagal, Kathmandu, Nepal; 2 Deurali Primary Health center, Nuwakot, Nepal

**Keywords:** *medical students*, *Pittsburgh sleep quality index*, *sleep quality.*

## Abstract

**Introduction:**

Medical students are under constant stress due to demanding academic load, fear of exam failure and hectic schedules. These factors can lead to poor sleep quality among medical students. Sleep quality of medical students not only determine their academic performance but is also important in determining long term effect on cognitive, psychosocial, behavioural as well as physical health of individuals. Although there are not enough recent studies to assess sleep quality of students, it is necessary to evaluate the condition of sleep among students. This study aims to find out the prevalence of poor sleep quality among medical students.

**Methods:**

This descriptive cross-sectional was conducted among undergraduate medical students of Kathmandu Medical College from October to November 2019 after taking ethical clearance from Institutional Review Committee of a tertiary care hospital before collecting data from participants. Subjects were recruited by simple random sampling from students of first, second, third and final years and were asked to fill the self-reported questionnaires, using Pittsburgh Sleep Quality Index. Descriptive statistical analysis was done using Statistical Software for Social Sciences version 24.

**Results:**

Out of 217 selected medical students, 96 (44.23%) of students have poor sleep quality with prevalence among male and female students as 41 (39.8%) and 55 (48.2%) respectively. The mean duration of sleep among students was 6.7±1.6 hours.

**Conclusions:**

Signifcant numbers of medical students have poor sleep quality which may affect their academic performance and may have long term impact on their health. Efforts must be directed towards educating about the sleep hygiene as well as proper time management skills.

## INTRODUCTION

Medical students are especially vulnerable to poor sleep quality.^1^ Sleep related disorders are prevalent in high number among medical students throughout the world.^2,3^ Sleep is a very important parameter for the consolidation memories and that being sleep deprived reduces one’s ability to learn.^4^ Quality of sleep can be categorized into subjective and objective aspects.^5^

Prevalence of poor sleep quality is higher among undergraduate medical students compared to students from other fields.^1^ Poor sleep quality effects academic performance and also effects intelligence and cognitive functions which is key for medical professionals.^6^ In Nepal, medical students have poorer attitude of sleep hygiene.^7^ Considering the consequences and prevalence of the problem, it is essential to determine quality of sleep among medical students.

This study aims to find the prevalence of poor sleep quality among medical students of Kathmandu Medical College.

## METHODS

A descriptive cross-sectional study was done on undergraduate medical students of Kathmandu Medical College (KMC) from October to November 2019. Ethical clearance was obtained from Institutional Review Committee (IRC) of Kathmandu Medical College before collecting data from participants. List of all the medical students who were present at the duration of study was obtained from college administration and consecutive number was given to the list. Simple random sampling technique was applied by using Random table to select study participants. Those students who were absent from regular classes for at least a month as well as interns were excluded from the study.

Sample size was calculated as:

n = z^2^ × (p × q)/e^2^

   = 1.96^2^ × (0.5 × 0.5)/ (0.05)^2^

   = 384

Where,

n = Sample size
z = 1.96 at 95% CI
p = prevalence of poor sleep quality among medical students (50%)
q = 1-p
e = margin of error, 5%

Adjusted sample size

   = n/(1+((n-1)/N))
   = 207
Where, N = Target population = 450

Therefore, the calculated samples size was 207 and considering 5% of non-response rate the sample size taken was 217.

All the respondents were informed about the aim and objectives of the study and informed consent was taken prior to study and they were aware that their participation was voluntary. Confidentiality of participants were ensured. Students were asked to fill up self-reporting questionnaire using Pittsburgh Sleep Quality Index (PSQI).^8^ Descriptive statistical analysis was done using Statistical Software for Social Sciences (SPSS) version 24.

## RESULTS

The prevalence of poor sleep quality among medical students was found to be 96 (44.23%) with Global PSQI score of more than 5. Out of 217 participants, 107 (47.5%) were male and 114 (52.5%) were female, among them 55 (25.3%), 36 (16.6%), 60 (27.6%) and 66 (30.4%) were studying in first, second, third and final year respectively. The average age of students was 21.39±1.6 years. Among male students, the prevalence of poor sleep quality was 41 (39.8%) which is lesser than female students with prevalence of 50 (48.2%) (Table 1).

**Table 1:** Sleep Quality Variation in Gender and Years of study.

Sex	Normal sleepers	Poor sleepers
Male	62 (60.2%)	41 (39.8%)
Female	59 (51.8%)	55 (48.2%)
Year of study		
First	32 (58.2%)	23 (41.8%)
Second	28 (77.8%)	8 (22.2%)
Third	28 (46.7%)	32 (53.3%)
Fourth	33 (50%)	33 (50%)

Poorer sleep quality is observed among the medical student staying at hostel 57 (47.9%) than those who doesn’t stay at hostel 39 (39.8%) (Table 2).

**Table 2:** Comparison of students who staying and not staying at hostel.

Staying in Hostel	Normal sleepers	Poor sleepers
Yes	62 (52.10%)	57 (47.9%)
No	59 (60.20%)	39 (39.8%)

The average duration of sleep among students was 6.7 ± 1.6 hours (Table 3).

**Table 3:** Sleep Duration.

Sleep duration	No. of students
More than 7 hours	78 (35.9%)
6-7 hours	93 (42.9%)
5-6 hours	33 (15.2%)
Less than 5 hours	13 (6%)

**Figure 1:**
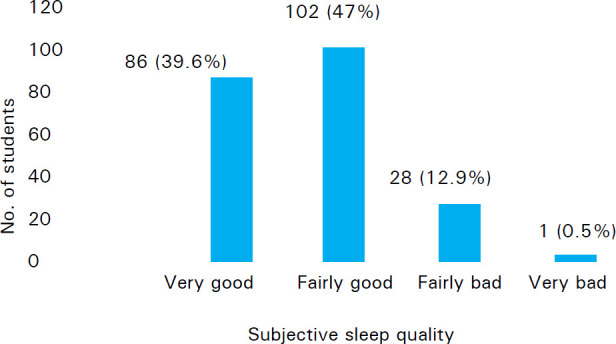
Subjective Sleep Quality.

Out of 217 students, 204 (94%) did not use sleep medications in the past one month whereas 13 (6%) used sleep medications at least once in the past one month. When students were asked that how much it took them to fall asleep, 137 (63.1%) students respond that it took less than 15 minutes whereas 80 (36.8%) respond it took them more than 15 minutes to fall asleep. Subjective sleep quality of most of the student 102 (47%) have fairly good sleep quality (Figure 1).

## DISCUSSION

Prevalence of poor sleep quality among students in our study is found to be 44% which was higher than a previous study done among college students using the same PSQI questionnaire is 35.4% though the study was conducted among undergraduate students non-medical study.^9^ The cause of poor sleep quality among medical students can be attributed to hectic schedule in medical college and increased academic load. Similar study done on medical student of Karachi, Pakistan showed prevalence of poor sleep quality of 39.5%, more prevalent female poor sleepers 44% than male 32.8% which is almost similar to our study.^10^

Sleep quality was poorer among students studying in clinical sciences (third and fourth year) in comparison to those studying in basic sciences (first and second year) may be due to comparatively more hectic schedules due to clinical rotations in clinical sciences in contrast to the study by Camila de Castro Corrêa et al. in which sleep quality is poorer in first and second year than third, fourth, fifth and sixth year.^11^ Poor sleep quality was found to be more prevalent among students staying in hostel rather than those stayed at home. This variation can be explained by late night social gatherings in the hostel as well as increased participation in extracurricular activities.

Our study was carried using a self-reporting questionnaire which is not quantitative assessment of sleep quality. Accurate assessment of sleep quality requires extensive tests such as polysomnography.

This study was conducted a single institution so, the findings of this study cannot be generalized and more studies at other medical institutions are required to find out actual prevalence of poor sleep among medical students.

## CONCLUSIONS

Every two out of five medical students found to have poor sleep quality which not only affecting their academic performance but also effect their physical health. This suggest the need of measures to identity the students at risk and educating medical students about proper sleep hygiene and the consequences of poor sleep practices.
